# Factors Impacting the Reduction in Neophobia Prevalence in Phenylketonuria Patients

**DOI:** 10.3390/nu16060768

**Published:** 2024-03-07

**Authors:** Meda-Ada Bugi, Iulius Jugănaru, Raluca Isac, Iulia-Elena Simina, Andrei-Ioan Munteanu, Niculina Mang, Georgiana-Flavia Brad, Delia-Maria Nicoară, Daniela Cîrnatu, Otilia Mărginean

**Affiliations:** 1 Ph.D. School Department, ‘Victor Babeş’ University of Medicine and Pharmacy of Timisoara, 300041 Timisoara, Romania; bugi.ada@umft.ro (M.-A.B.); nicoara.delia@umft.ro (D.-M.N.); 2 Department of Pediatrics I, Children’s Emergency Hospital ‘Louis Turcanu’, 300011 Timisoara, Romania; juganaru.iulius@umft.ro (I.J.); munteanu.andrei@umft.ro (A.-I.M.); mang.nina@umft.ro (N.M.); brad.giorgiana@umft.ro (G.-F.B.); marginean.otilia@umft.ro (O.M.); 3 Department of Pharmacy, University of Medicine and Pharmacy ‘Vasile Goldis’, 310025 Arad, Romania; 4 Department XI Pediatrics, Discipline I Pediatrics, ‘Victor Babeş’ University of Medicine and Pharmacy of Timisoara, 300041 Timisoara, Romania; 5 Department XI Pediatrics, Discipline I Pediatrics, Disturbances of Growth and Development in Children–BELIVE, 300011 Timisoara, Romania; 6 Department of Pediatrics II, Children’s Emergency Hospital ‘Louis Turcanu’, 300011 Timisoara, Romania; isac.raluca@umft.ro; 7 Department of Pediatrics, IIIrd Pediatric Clinic, ‘Victor Babeș’ University of Medicine and Pharmacy of Timisoara, 300041 Timisoara, Romania; 8 Department of Genetics, Center of Genomic Medicine, ‘Victor Babeş’ University of Medicine and Pharmacy of Timișoara, 300041 Timisoara, Romania; simina.iulia@umft.ro; 9 Department of Medicine, University of Medicine and Pharmacy ‘Vasile Goldis’, 310025 Arad, Romania

**Keywords:** neophobia, phenylketonuria, nutrition

## Abstract

Food neophobia (FN), the fear of sampling new foods, can have a significant impact on children’s eating habits. Children with phenylketonuria (PKU), a hereditary condition that inhibits the body’s capacity to metabolize phenylalanine, should take this attitude with caution. Patients with PKU must follow a rigorous phenylalanine (Phe)-restricted diet to avoid brain malfunction that can include intellectual disability, seizures, and behavioral difficulties. The novelty of our work stems from the fact that we explored the origins of this incorrect intake pattern, which exacerbates PKU patients’ already fragile health. We conducted a cross-sectional study on 34 previously diagnosed phenylketonuria patients and a control group ranging in age from 7 months to 40 years, with a sex ratio of M/F 2:1. The Food Neophobia Scale (FNS) was used to determine neophobia. We used JASP (version 0.18.1) statistical analysis to examine the relationship between neophobia and PKU condition, age and nutritional status at the time of study, diet compliance, parental educational level, period from birth to PKU diagnosis, and environmental (rural/urban) provenience of PKU patients. According to the data, 61.76% of patients with PKU were neophobic, as were 70.57% of the control group. Food neophobia was associated with PKU patients’ present age, the period from birth to PKU diagnosis, and parental educational level.

## 1. Introduction

Phenylketonuria (PKU; OMIM 261600) is a rare autosomal recessive genetic disorder triggered by mutations in the phenylalanine hydroxylase (*PAH*) gene, leading to reduced catalytic activity and affecting the breakdown of phenylalanine (Phe) [1]. The excess of Phe causes severe and irreversible intellectual disability, along with autistic behaviors, motor impairments, rashes, and seizures [2]. At present, there is no curative treatment for PKU. The primary approach to managing the condition predominantly involves strict dietary control. Dietary Phe intake should be minimized to meet the essential requirements for normal growth, supplemented with specially created metabolic formulas. The initiation of this regimen is recommended in infancy, ideally within the five days post-birth [3]. Adherence to the prescribed diet is important as failure to do so may render the severe neurological impairment irreversible [4]. The highest reported prevalence of PKU was found in Turkey; 38 per 100,000 neonates were diagnosed with PKU, based on data from 46 research studies conducted between 1964 and 2017. Conversely, the lowest prevalence was obtained in Thailand; 0.3 per 100,000 neonates suffer from PKU [5]. Based on the previously mentioned sources, it has been stated that the worldwide incidence rate of the disease stands at 6 cases per 100 infants. The forecast prevalence rate in Romania is 1 case per 100,000 individuals [6]. Dietary restrictions observed in PKU patients’ diets bring to the surface the most common attitudes related to novel food acceptance: neophobia, picky/fussy, and neophilia. Food neophobia (FN), defined as the aversion or reluctance to consume unfamiliar foods, is a psychological phenomenon that must be distinguished from pickiness, which is the refusal to consume familiar foods that are detested [7,8,9]. To date, our comprehension of the relationship between food neophobia in individuals with PKU and various clinical and treatment-related factors, such as age, blood Phe control, anthropometric measurements, lifestyle choices, breastfeeding during infancy, and the timing of introducing solid foods, remains limited [10]. Within the confines of these dietary restrictions, it is essential to promote normal consumption behavior and a wide variety of flavors in order to maximize food options [11]. The early administration of L-amino acid supplements with a bitter flavor to neonates with PKU may influence the development of food preferences [12]. In addition, there is evidence that such early flavor experiences have a lasting impact on flavor preferences [12,13,14]. Therefore, the precise impact of food neophobia in PKU has yet to be thoroughly investigated, especially in relation to the introduction of L-amino acid supplements and their potential long-term effects on food preferences [12,13,14,15]. Food neophobia significantly impacts the dietary choices and overall daily routines of children and caregivers [16]. The phenomenon in question holds evolutionary relevance, yet it can give rise to avoidance habits and present challenges for youngsters who adhere to dietary restrictions [17,18]. It falls under avoidant/restrictive food intake disorder and affects psychological well-being due to taste familiarity and parental attitudes [18,19]. Multiple factors contribute to food neophobia, necessitating countermeasures against its influence on children’s preferences and nutritional imbalance [19]: dietary diversity, health, development, body mass index (BMI), age, diagnostic moment, and parent’s level of education [20]. The objective of this study was to examine the relationship between neophobia and socioeconomic status, familial background, or dietary habits among patients with PKU.

## 2. Materials and Methods

### 2.1. Study Population

We conducted a cross-sectional study between 2018 and 2023 using self-applied online questionnaires on individuals previously diagnosed with phenylketonuria, including those under the care of the “Louis Turcanu” Emergency Hospital for Children in Timisoara or affiliated with the PKU Life Romania Association or their parents. The inclusion criteria were a prior diagnosis of PKU. Of the 70 patients who met the inclusion criteria, 34 agreed to participate and were analyzed in this study as the PKU group (PKUG). The sample comprised an equal number of non-PKU controls, carefully selected to match in terms of both age and sex. The control group (CG) consisted of patients admitted to the Pediatric Ward of the Timisoara “Louis Turcanu” Emergency Children’s Hospital. Regarding age, the patients were further divided into five categories: <2 years (8.82%), 2–7.9 years (44.11%), 8–13.9 years (17.64%), 14–17.9 years (17.64%), and ≥18 years (11.76%).

The research adhered to the guidelines outlined in the Declaration of Helsinki and Resolution 466/2012 of the National Health Council. The research protocol received approval from the Ethics Committee of the “Victor Babes” University of Medicine and Pharmacy (No.60/12.11.2018) and from the hospital’s Ethics Committee (No.3392/24.02.2023). Additionally, each participant provided a signed informed consent.

### 2.2. Data Collection

An interview questionnaire partly developed by the researcher after reviewing the related literature and translated into the Romanian language was used to carry out the current study and offered to the participants. The respondents filled in the online survey using the Google Forms platform. The questionnaire consisted of three parts: the participant’s characteristics, familial characteristics, and eating behavior. The first part comprised the subject’s identifying information, including age, sex, anthropometric measurements at birth and during data collection, PKU diagnosis details (year and method of diagnosis—neonatal screening through dry blood spot or other method), and Phe level in µmol/L at the diagnosis. To assess the nutritional status of pediatric patients, percentiles were utilized, and specific terminology was employed, interpreted by referencing World Health Organization girls’ percentiles expanded tables [21] and boys’ percentiles expanded tables [22]. The second part included familial characteristics, such as the socio-economic status of the family, the educational level of the parents or guardians, and specifications regarding the person taking care of the child and preparing/supervising the diet. The third part comprised details regarding the eating behavior, in which the child’s eating habits were assessed using the Food Neophobia Scale (FNS) [6]. The specific measurement of food neophobia in adults commonly employs the FNS, a validated questionnaire consisting of ten items and developed by Pliner and Hobden. Comprising 10 items, the scale includes 5 related to neophobic behavior and 5 related to neophilic behavior. A higher FNS value indicates a greater inclination toward neophobia. This scale proves valuable for measuring the willingness to try new food items, investigating the acceptance of exotic cuisines, and exploring expectations regarding novel food items [23]. We chose to apply this questionnaire to both adults and children, translating the 10 statements to Romanian and requesting parents to help their children or to answer on their behalf when/if necessary. The questionnaire was applied to a CG, respecting the sex ratio and distribution by age groups. The typical Likert scales consist of seven choices. However, this configuration poses certain challenges. The inclusion of a “neutral” option may lead survey respondents to easily bypass the question, potentially choosing this middle ground without much deliberation. In contrast, a six-point scale encourages participants to approach the question with greater consideration, compelling them to make a choice that leans either positively or negatively [24]. Given that our perceptions often lean away from neutrality, we considered the seven-point scale to better capture the nuances of the subjective experiences of our respondents. They were asked to answer each statement on a Likert scale of 1–7, where 1 stood for “strongly disagree”, while 7 stood for “strongly agree”. Regarding statement translation, we have replaced the term “ethnic” with “specific” as it includes references to restaurants or cuisines of different cultures and countries, proven to influence neophobia [23,24,25,26].

The individual total scores were calculated by summing the values assigned to each scale item, ranging from 1 to 7, resulting in a total score range of 10 to 70 points. The statements used in this study and their corresponding English translations are noted in Supplementary Table S1. Therefore, an increase in the total individual score signifies a food neophobia level. Similar to previous research studies [25,26,27,28], the participants were categorized into three groups based on their FNS total scores, using cutoff points defined as the FNS total scores ± standard deviation: neophilic, neutral, and neophobic groups. Despite the lack of a standardized approach for setting FN cutoffs, we categorized patients as neutral (FN score = 30–35), neophobic (FN score > 35), or neophilic (FN score < 30). Additionally, the results of the FNS were compared between the two groups.

### 2.3. Data Analysis

The distributions of collected data analyzed with the Shapiro–Wilk test were shown to be not normally distributed for birth height (cm), current age (month), BMI, current height (cm), current level of Phe (µmol/L), current weight (kg), year of diagnostic phenylketonuria, diagnostic year (month), and level of Phe at birth (µmol/L). Consequently, a nonparametric Mann–Whitney U test was used for intergroup comparison, while Pearson’s r test was used for correlations. For the Mann–Whitney test, the effect size was given by the rank biserial correlation. All statistical analyses were performed using the JASP (Version 0.18.1 [29]).

### 2.4. Hypothesis

In order to assess the factors impacting the reduction in neophobic prevalence in phenylketonuria patients, the following hypotheses were formulated:

**H1.**  *There is a distribution variation for FN score among PKU and control groups.*

**H2.**  *There is a relationship between the patient’s current BMI and the development of neophobia.*

**H3.**  *There is a relationship between diet compliance and neophobia.*

**H4.**  *There is a relationship between a patient’s Phe level at the diagnostic moment and the development of neophobia.*

**H5.**  *There is a correlation between parental educational level and the patient’s development of neophobia.*

**H6.**  *There is a correlation between the patient’s age and the onset of neophobia.*

**H7.**  *There is a relationship between the length of the period from birth to diagnosis and neophobia.*

**H8.**  *There is a variation of distribution for FN scores among urban and rural patients.*

## 3. Results

Sixty-eight participants (34 PKU patients and 34 controls) were included in the study. The sex ratio M/F = 2:1, and the age groups between the PKU group and the CG were comparable (Table 1). Patients with PKU were diagnosed in the following ways: 29.41% within the first 15 days of life, 44.11% between days 16 and 30, 14.70% between day 31 and three months, 2.94% by the end of the first year, and 8.82% beyond the first year. Of the patients, 88.23% were diagnosed using screening. The individuals within the PKUG had a substantial prevalence of malnutrition, with 35.30% displaying underweight, 23.53% being overweight, and 23.53% displaying severe obesity.

Table 2 provides a summary of the *PAH* gene mutations observed in patients for whom genetic results were available. Notably, all patients exhibited pathogenic mutations, with 29.4% identified as heterozygotes. Among these individuals, three manifested classic or moderate phenotypes of PKU, while only one displayed a mild phenotype of the disorder.

After the questionnaire’s data were analyzed, we observed that an important percentage of both PKUG and CG respondents had FNS scores exceeding 35. For instance, in the over-18-year-old group, the percentage was 100% for all the subjects. Table 3 provides a summary of the data for each age group, with further division based on sex. There were no statistical differences between incidences of neophobia by sex.

**Figure 1:**
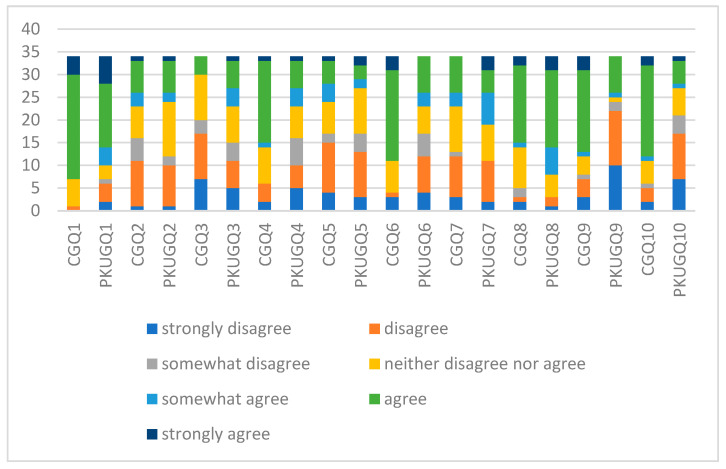
Responses to the food neophobia questionnaire using the Likert scale.

The answers to each question revealed significant disparities for several questions—Q4, Q6, or Q9—even though the FNS scores were rather close. The outcome of each question is shown in Figure 1 for both groups.

**Figure 2:**
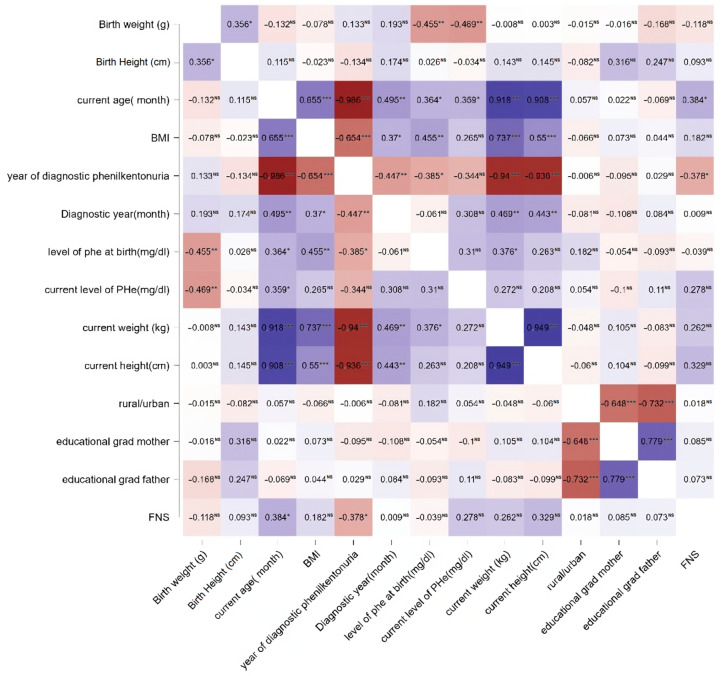
Statistical correlations among analyzed variables. Significance levels: *** *p* < 0.001, ** *p* < 0.01, * *p* < 0.05. Nonsignificant correlations are marked as ‘NS’. The color gradient in the figure reflects the strength of significance, with deeper shades representing more substantial associations, and lighter tones suggesting comparatively lower statistical significance. Positive correlation coefficients denote positive associations, while negative correlation coefficients indicate negative associations.

The results of the statistical tests employed for hypothesis testing are presented in Figure 2. The data show statistically significant correlations between the score on the FNS (Food Neophobia Scale), the current age, and the period between birth and diagnosis. Consequently, as age advanced, the FNS evaluation score tended to be higher, indicating an increased risk of neophobia with age. A correlation between the two was observed regarding the duration between birth and diagnosis, such that an earlier diagnosis was associated with a lower risk of obtaining a high score on the FNS evaluation scale.

We applied additional tests for the observed correlations to corroborate the findings and provide an enhanced statistical data analysis. The parents’ levels of education and the distribution of the PKU group’s scores were the subjects of the further tests. Table 4 presents a comparison of data between PKUG and CG, encompassing factors such as development, FNS, the primary caregiver’s educational level, and the level of Phe at birth and currently. The results revealed statistically significant differences in the primary caregiver’s educational level and, to some extent, in the FNS scores between groups.

Given the findings that highlighted a significant variation in the parents’ educational levels based on whether they came from urban or rural environments, a follow-up inquiry was conducted. A comparison of the outcomes of the correlation between the FNS score and each parameter investigated by PKUG based on the environment of origin (rural/urban) is presented in Table 5. The results showed no significant differences for any of the investigated items.

Synthesizing the results of the statistical analyses from the eight proposed hypotheses, only three were confirmed: those related to the correlations between FNS and age and the correlations between FNS and the interval between the moment of birth and the diagnosis of PKU, as seen in Table 6.

## 4. Discussion

While food neophobia unquestionably shapes the initial encounter with novel foods, it is crucial to recognize that the decision to consistently include such foods in one’s diet is influenced by a complex interaction of additional factors [31]. The concept of “picky/fussy” behavior is distinct from food neophobia [8]; however, measures regarding these attitudes are still in development [32]. “Picky/fussy” individuals are particularly children who consume an insufficient variety of foods by rejecting both familiar and unfamiliar foods [33]. Concurrently, the term “neophilic” refers to a characteristic in which an individual or group demonstrates a strong propensity and willingness to experiment with new foods. Neophilic individuals or cultures are inclined to embrace culinary innovations; investigate a variety of ingredients, flavors, and dishes; and be willing to experiment with unconventional food pairings [34]. This disposition contributes to the variety of culinary practices, the adaptability of the diet, and the incorporation of novel nutritional sources. Of the three attitudes toward new foods, neophobia has the most significant impact on the dietary choices of PKU patients. In this study, a high prevalence of food neophobia was observed in both the PKU patient group and the control group. This conclusion was not surprising given that the majority of the participants in our study were children. Previous research has consistently shown a higher prevalence of neophobic behavior among parents of children in both the PKU group and the control group, compared with adults [3,35]. According to our results, food neophobia does not seem to be associated with BMI, the phenylalanine level at the time of diagnosis, parental education level, or the patient’s background. Instead, food neophobia appears to be more influenced by the patient’s age and the year of PKU diagnosis. Our study found no association between patient’s sex and FNS, consistent with previous research [36]. A study from 2016 carried out in Germany that focused on adolescents found no significant difference in food phobia levels between boys and girls [37]. However, the researchers highlighted that food neophobia levels in relation to sex were influenced by age. Despite achieving optimal metabolic control, the current Phe level, characterized by maintaining phenylalanine levels within the established reference range, did not play a significant role in fostering a higher aversion or reluctance toward experimenting with novel foods. Similar results were revealed in a previous study by Tonon et al. [10]. With the introduction of a modified diet in 1951, newborn screening for PKU was initiated, allowing affected individuals to live normal lives [5]. Despite the difficulty of adherence, phenylalanine restriction in the diet has been the primary treatment for phenylketonuria for over 60 years [7]. Diets low in phenylalanine and novel drug mechanisms [14] keep blood phenylalanine concentrations within the target range. However, the diet’s lifetime adherence is unknown, and the organism’s protein status may be compromised [9]. Compliance with the restrictive diet is challenging, and abandonment can lead to a decline in academic performance and psychosocial problems [9]. The management of PKU requires adherence to a phenylalanine-restricted diet and routine clinic visits, presenting patients with ongoing challenges and negatively impacting their quality of life [12]. Increasing natural protein intake while maintaining metabolic control may improve outcomes, but the optimal protein intake is unknown [13]. The strict dietary regimen substantially impacts the quality of life, causing a substantial proportion of patients to discontinue treatment. Dietary cessation can worsen symptoms and raise phenylalanine levels [27]. This dietary management approach necessitates significantly reducing high-protein foods, like meat, fish, eggs, cheese, nuts, seeds, and pulses. To compensate for the restricted protein intake, individuals with PKU are supplemented with bitter-tasting Phe-free L-amino acids. Consequently, the diet naturally becomes high in carbohydrates, particularly from plant and cereal sources, to fulfill energy requirements [9]. From an evolutionary standpoint, food neophobia might have conferred a selective advantage by safeguarding against harmful foods in patients with PKU or limits to dietary diversity and causes of malnutrition. Considering the significance of the phenylalanine-free diet in the progression of the disease, adherence to it is crucial for the lifelong treatment of PKU patients. Critical to the progression of the disease is the early detection of the medical condition at birth, continuous monitoring of patients, and providing support to both patients and their families. The neonatal screening system in Romania is based on collecting a single drop of blood 48–72 h after birth. Despite management guidelines for phenylketonuria recommending the initiation of dietary treatment between 7 and 10 days of life, our study indicates that a significant number of patients commence treatment at a later stage. In 2022, the number of children with PKU monitored was 342. Patients with phenylketonuria in Romania who are enrolled in the National Program for Women and Children have access to the following foods: protein substitutes, infant formulas without phenylalanine, and hypo protein dietary products with reduced phenylalanine content (such as flour, cereals, pasta, biscuits, semolina, egg replacer, and cooking mixes) are available for those with phenylketonuria. Foods intended for special medical use in the case of phenylketonuria are administered under strict medical supervision and are tailored to individual cases, tolerance, weight, age, and body size. The neonatal screening situation in Romania can be compared with that of other European countries and the rest of the world in the EAEC regarding coverage, the number of conditions screened for, and screening methods. In France, for instance, an extended test is administered for approximately 50 conditions, including congenital diseases of metabolism, endocrine disorders, and hematological disease [4]. In the United Kingdom, the national neonatal screening program searches for a variety of conditions, including congenital hypothyroidism, phenylketonuria, and cystic fibrosis.

Analyzing the interval between birth and diagnosis, we observed that one of the subjects received a diagnosis at the age of 10 years. For statistical interpretation, this value with an abnormal distribution was excluded. The mean interval until the diagnosis was 21 days, whereas guidelines recommend an interval of up to 10 days. Our analysis aimed to explore whether the age of patients with PKU can indicate a tendency toward food neophobia. Establishing a diet low in phenylalanine from birth, diversifying appropriately to prevent micronutrient deficiencies, and maintaining a regimen meeting the individual’s micronutrient requirements throughout life are all necessary. From this perspective, an early diagnosis and the implementation of an appropriate diet to reduce the risk of metabolic imbalance are essential. However, another nutritional condition, pickiness, exists in patients younger than 5 years old [38]. High scores on the FNS suggest a reduced anticipation of liking unfamiliar foods, limited familiarity with foreign cuisines [1], and a decreased willingness to try new and unfamiliar foods. Pliner described how differences in measured food neophobia did not significantly correlate with actual hedonic responses when participants tasted unfamiliar foods. However, other studies have indicated that individuals with higher levels of food neophobia tended to express less liking for unfamiliar foods compared with those with lower neophobia [5,6]. It has been observed that early exposure to a particular food significantly enhances the willingness to eat that food again, regardless of the individual’s level of food neophobia [4]. This suggests that food neophobia primarily influences responses to unfamiliar foods rather than familiar ones [1,6]. It is evident that food neophobia plays a role in shaping the initial tasting experience of unfamiliar foods. However, the decision to continue consuming such foods is influenced by a combination of other factors as well [6].

Nutritional support is required for the duration of a person’s life; however, support is also required to perceive treatment as a positive factor, particularly during adolescence [39]. Lifelong maintenance of blood phenylalanine concentrations within the therapeutic range is a challenging objective. It is not uncommon for adults with PKU to discontinue their dietary management [40]. During adolescence and adulthood, when many individuals with PKU find the restricted diet disagreeable, difficult to follow, and a hindrance to social relationships [41], therapeutic phenylalanine levels may be difficult to achieve. Previous research has demonstrated that a restrictive diet does not hinder physical development [42]. All children who switch from specific infant feeding to foods consumed by family members might have difficulties embracing new foods, not just PKU patients, as a potential natural developmental stage [17].

Despite recommendations to maintain blood phenylalanine concentrations in the therapeutic range throughout life, it is not uncommon for adults with PKU to discontinue dietary management of their disorder. An early diagnosis was associated with a reduced need for special education or other special services, and continuous treatment was associated with decreased psychological co-morbidities. In our study, preschooler-age children’s average food neophobia score was 35.3, which was higher than the results obtained in another study, 23.73 ± 4.45 (25). In the same study, parental modeling (β: −0.470; 95%CI: −0.732, −0.207) and the frequency of children eating with their families at home (β: −0.407; 95%CI: −0.707, −0.108) were negatively associated with children’s food neophobia scores [43]. A previous study showed there was a statistically significant correlation between the neophobia that children develop and the neophobic characteristics of their parents’ dietary behavior [44]. Pliner found that variations in quantified food neophobia did not correlate significantly with actual hedonic responses when participants sampled unfamiliar foods [1]. In contrast, other studies [45,46] have suggested that individuals with elevated levels of food neophobia tend to have a lower preference for unfamiliar cuisines than those with lower levels of neophobia. This observation suggests that psychological and sensory factors interact intricately to determine food preferences [19]. In addition, research has shown that early exposure to specific foods increases a person’s willingness to ingest them in the future, regardless of their initial level of food aversion [47]. This indicates that food neophobia affects responses to unfamiliar cuisines more than familiar ones [1,6]. For all the participants, the FNS score was calculated by adding the average score for each statement belonging to the FNS. The average FNS score was 30.35 on the PKUG and 44.09 in CG in this study and was compared with the FNS scores from other countries: 38.5 for Indonesia, 37.4 for the Philippines, 39.3 for Malaysia, 34.1 for Vietnam, 26.1 for the UK, and 34.7 for Australia [48]. It must be mentioned that our subjects are primarily children, which could explain the high numbers on the FNS. The incidence of obesity in our study group was comparable to that in the general population, as demonstrated by a previous meta-analysis that found no evidence to support the notion that a Phe-restricted diet is a risk factor for overweight or obesity [4]. One longitudinal study found that food neophobia at 1 year was positively associated with the later introduction of dairy products, the use of ready-prepared baby foods, and the use of ready-prepared adult foods [49]. In our study, diet adherence did not affect the FNS, which is similar to age, BMI, and sex. Identical results were discovered by other researchers as well [50,51,52].

To ascertain whether the genetic phenotype had an impact on the FNS score, we analyzed the available genetic mutations in our patients. The majority of patients exhibited classic phenotypes of PKU, marked by the presence of either heterozygous or homozygous mutations within the *PAH* gene. Consistent with findings in the literature [53,54], the p.Arg408Trp mutation emerged as the most prevalent among these cases, highlighting its role in the pathogenesis of PKU. Our study also revealed a subset of individuals presenting with moderate or mild phenotypes of the disorder, but their number was too small to be able to make assumptions regarding the influence of the genetic phenotype on the FNS score.

When considering each question in the form, regarding Q1, the control group had a more significant percentage of people who “strongly agree” and “agree” to try new foods (79.41%) than the PKU group (58.82%). This variation might occur because individuals in the control group have fewer dietary limitations than those in the PKU group. Being on a specialized diet, the latter group must exercise greater caution in their food choices, contributing to this distinction. Analyzing respondents who would “strongly disagree” and “disagree” with trying new foods, the percentage is 2.94% in the control group and 17.65% in the PKU group. This reinforces the reluctance of PKU patients to try new foods. Previous investigations in the PKU group found that neophobia was a significant factor in food refusal [35]. Regarding the PKU group, however, many respondents attempt new foods less frequently or even avoid them. This is well known in the literature even from 2007, when researchers [9] pointed to food neophobia as an evolutionary safeguard to prevent humans from consuming potentially hazardous food. Therefore, it is not unexpected that within our study, individuals with PKU exhibited neophobic tendencies [55]. Variable responses to continually trying new foods may indicate divergent approaches to accepting and incorporating food diversity into a limited diet among PKU patients. It is essential to recognize that strict diets and the need to monitor phenylalanine levels can affect the willingness to try novel foods.

For the second question, the PKU group showed a higher proportion (35.29%) of individuals who expressed a neutral stance (neither disagree nor agree) toward trying new foods compared with the control group (20.59%). This variance may be attributed to the dietary constraints imposed on individuals with phenylketonuria (PKU), necessitating adherence to a stringent diet. As a result, their curiosity toward unfamiliar foods must be regulated to prevent health complications. Many respondents lack confidence in novel foods, indicating a lack of faith in their quality or safety. The propensity of PKU patients to rely on foods with known and monitored phenylalanine content may be reflected by a lack of confidence in ingesting novel foods. This reaction can be interpreted as a preventative measure to avoid accidentally consuming foods that could alter the nutritional balance specific to PKU.

The divergence in responses for Q3 between the control group and the PKU group regarding trying food without knowledge of its ingredients highlights distinct perspectives shaped by different priorities. Within the control group, a significant 50% expressed strong disagreement or disagreement with the notion, suggesting a prevalent willingness to explore food even without comprehensive ingredient knowledge. This openness reflects a curiosity and willingness to embrace new culinary experiences. Conversely, in the PKU group, where strict dietary control is crucial due to health concerns, approximately 32.35% disagreed with trying food without prior knowledge of its ingredients. This inclination aligns with the necessity for careful dietary management among individuals with PKU. These contrasting percentages underscore the impact of health considerations and diverse attitudes on food choices. While a considerable portion of the general population remains open to culinary exploration despite limited ingredient awareness, the PKU group’s response emphasizes the importance of health-related caution in food consumption. The majority of participants appear to avoid foods whose ingredients are unknown. This may be indicative of a circumspect approach to unfamiliar ingredients. Observing that unfamiliar foods should be avoided has significant implications for PKU patients. This behavior may be determined by the need to know the exact amount of phenylalanine in the food ingested to avoid excessive consumption.

In the study’s Question 4 responses, it becomes evident that a more significant percentage of individuals in the control group (55.88%) agreed with trying food from other countries compared with the PKU group (20.59%). This disparity can be attributed to the absence of strict dietary restrictions within the control group, allowing them the freedom to explore and sample a more comprehensive array of foods from various countries. The majority of participants did not appear to be interested in foreign cuisine. However, a significant number of participants were either less or more willing to try foods from diverse cultures.

In Question 5, a higher percentage of individuals in the control group (44.12%) expressed disagreement and strong disagreement toward trying foreign food due to its perceived extreme dissimilarity, compared with the PKU group (38.24%), which also demonstrated a noteworthy percentage. This variance might be clarified by the tendency of individuals in the control group to emphasize the appearance aspect of a new food. Patients with phenylketonuria may have a range of preferences and comfort levels regarding culinary exploration, as indicated by this diversity of attitudes. The interest in cuisines from other countries suggests that PKU patients may be positioned favorably toward opportunities for dietary diversity. Due to their familiarity with dietary restrictions, their appreciation of cultural diversity may reflect a desire to investigate alternative and safe sources of nutrients. A substantial proportion of participants were unwilling to sample ethnic foods, possibly because they viewed them as “strange” or “unusual”. Patients with phenylketonuria are conditioned to avoid novel and unusual foods based on their phenylalanine content, which may contribute to their negative perceptions of ethnic food. This can result in an unwillingness to experiment with foreign flavors and ingredients.

When discussing the exploration of new foods (Q6), particularly at parties, the contrast between the PKU group and the control group was striking. In the control group, a considerable 67.65% expressed agreement or strong agreement with the idea of trying new foods. This suggests a prevalent openness and willingness to indulge in culinary experiences. Conversely, within the PKU group, only 23.53% shared this sentiment, indicating a substantial divergence in attitude. This discrepancy underscores the necessity for individuals with PKU to be exceptionally cautious and vigilant when navigating unfamiliar foods. The stringent dietary restrictions imposed by PKU make it imperative for these individuals to exercise careful consideration and scrutiny regarding the ingredients before trying new dishes, particularly in social settings like parties. These percentages underscore the distinct challenges faced by the PKU group, emphasizing the need for heightened caution and conscientiousness due to the health-related dietary constraints imposed by their condition. This stark response highlights the unique considerations shaping the relationship between individuals’ health conditions and their approach to trying new foods, especially within social contexts. It appeared that participants were more willing to sample new foods at dinner parties, indicating a greater social willingness to experience food. The ability to overcome food neophobia is significantly influenced by social factors, as evidenced by the willingness to sample new foods at dinner parties. The social context can facilitate the development of a framework of approval and encouragement for culinary experimentation.

In the seventh question, noteworthy percentages in the control group were 29.41% expressing neutrality, while 23.53% provided a neutral response in the PKU group. This contrast might be elucidated by the comparative absence of stringent phenylalanine intake regulations in the control group, affording them greater assurance when encountering unfamiliar studies. Significant numbers of participants and their children feared eating unfamiliar foods, indicating a certain degree of apprehension in the face of novel culinary experiences. PKU patients’ constant awareness of the potential impact on their phenylalanine levels may contribute to their irrational aversion to foreign cuisines. This demonstrates a delicate balance between the desire to try novel foods and the need to maintain strict dietary control.

In assessing children’s food selectivity, both the control and PKU groups showed a 50% similarity, indicating comparable discernment levels. However, this alignment is not solely due to PKU-related dietary restrictions. In the control group, selectivity might be due to taste preferences or habits, not just health concerns. Differences arose in the middle ground: 26.47% in the control group vs. 14.71% in PKU. Interestingly, while only 2.94% in the control group somewhat agreed, 17.65% in the PKU group leaned toward agreement. This suggests a wider openness among PKU individuals to potential food choices compared with the control group, emphasizing varied influences on their preferences. Participants’ food preferences appeared to span a broad spectrum, from moderate selectivity to greater openness. The stringent need to adhere to dietary restrictions can justify demanding food choices. Patients with PKU are aware of the significance of nutritional balance and limiting phenylalanine intake so they can make more prudent food selections.

The responses for Q9 to the statement “My kid eats almost everything” revealed distinct attitudes toward children’s eating habits between the control group and the PKU group. In the control group, a significant 61.76% strongly agreed or agreed with this statement, indicating a prevailing belief that children within this group had versatile eating habits and were open to a wide variety of foods. This suggests a general perception of less selectivity in dietary preferences among children in this cohort. Conversely, within the PKU group, a notably lower 23.53% strongly agreed or agreed with the statement. This stark contrast likely stems from the stringent dietary restrictions imposed by PKU, leading to a more selective approach to food choices among individuals managing this condition. The higher percentage of disagreement in the PKU group (64.71%) further underscores the impact of these dietary limitations on their perception of a child’s eating habits. These considerable percentage differences between the two groups highlight the divergent attitudes toward children’s food acceptance. The control group’s higher agreement suggests a broader dietary openness among children. In comparison, the lower agreement in the PKU group underscores the influence of health-related dietary constraints on a child’s food choices and the heightened selectivity necessitated by the condition. Most respondents indicated they were willing to consume almost any variety of food, indicating that their palates were adaptable. The willingness to attempt new foods can be interpreted as a desire to consume all safe and permissible PKU diet foods. Within the limitations imposed by their medical condition, this approach reflects a favorable attitude toward nutritional diversity.

The marked contrast in responses between the control and PKU groups regarding their child’s preference for food at specific restaurants (Q10) showed significant divergence in perceptions and preferences. Within the control group, a substantial 64.71% expressed agreement (agree or strongly agree) with the statement, indicating a prevalent belief that children in this cohort were inclined to enjoy food at ethnic restaurants. This suggested an openness to cultural culinary experiences and a positive attitude toward diverse cuisines. On the contrary, in the PKU group, only 17.65% agreed with the statement. This substantial difference of 47.06 percentage points indicated a markedly lower inclination toward their child’s preference for specific restaurant food. The lower agreement percentage within the PKU group aligns with the dietary limitations associated with managing PKU, possibly resulting in a more cautious approach to diverse cuisines and ethnic restaurant foods. Additionally, the contrast in disagreement percentages is noteworthy. While 14.71% in the control group disagreed with the statement, a higher percentage, 50%, within the PKU group expressed disagreement. This significant discrepancy of 35.29 percentage points highlights a more pronounced opposition to their child preferring food at ethnic restaurants among the PKU respondents. These striking disparities underscore the profound impact of health-related dietary constraints on the perceived preferences of children in different culinary settings. The higher inclination toward ethnic restaurant food in the control group compared with the PKU group emphasizes the need to consider health conditions and their associated dietary limitations when evaluating preferences for diverse cuisines. The attendees’ willingness to try new specific restaurants demonstrates their appreciation for international culinary experiences. In the context of the restrictive PKU diet, dining at ethnic restaurants could be considered a pursuit for alternative dining experiences. In this context, specific restaurants can provide patients with the opportunity to experience a variety of flavors and ingredients in a controlled environment. Regarding the impact of food neophobia in patients with phenylketonuria, we can see how the constant focus on phenylalanine control can contribute to the tendency to avoid unfamiliar foods. This may provide a level of nutritional security, but it may limit opportunities to experience variety and culinary innovation. It is essential to investigate these relationships further and determine how to balance specific dietary requirements with the desire to diversify diets and try new foods safely.

Building upon our FNS results, which indicated that the primary caretaker was the mother in 91% of cases and that 47.1% of mothers had a university education level, our study highlights the significance of family dynamics and the educational level of caregivers in managing neophobia among patients with PKU. To address these factors, we propose targeted interventions. First, personalized guidance and resources should be provided to empower mothers, particularly those with higher education levels, to actively engage in dietary management strategies. Leveraging their knowledge and skills, mothers can play a pivotal role in optimizing dietary adherence and overcoming neophobic behaviors in their child with PKU. Furthermore, we advocate for the encouragement of active participation from both parents, with a focus on the primary caretaker (typically the mother), in meal planning, preparation, and food exploration activities. This collaborative approach within the family unit can foster a supportive environment and facilitate the successful implementation of dietary strategies to mitigate neophobia and promote overall well-being in patients with PKU.

As a notable observation, the FNS scores were higher in the control group compared with PKU patients. This discrepancy could be attributed to the study’s timing during the COVID-19 pandemic, leading to increased home-based eating habits among children. Additionally, concerns about accidentally consuming allergens in foods may have contributed to heightened fears among parents or children in the control group. Unfortunately, comprehensive information about the control group is limited to the details provided in the questionnaires.

The results have to be viewed in light of certain study limitations. First, the control group included children with different pathologies. Adult patients were enrolled from the nutrition clinic, indicating individuals in need of dietary habit changes. Another limitation of the study may be the diversity of ages accepted in the research since food neophobia at young ages can be confused with typical food preferences or whims. Additionally, our study is constrained by the limited availability of protein substitutes in Romania and the lack of data regarding the incorporation of glycomacropeptides into the diet within the Romanian National Phenylketonuria Program, as these were introduced in 2022. Specifically, for each age group, there are at most two options with similar characteristics, with a maximum difference of 5 g protein equivalent per 100 g product. Our study is also limited by the small number of participants and the short time frame during which they were observed. It is plausible for FN values to fluctuate with age. Therefore, multicenter prospective studies are warranted to enhance the generalizability of our findings and contribute to a more comprehensive understanding of the relationship between age and food neophobia in this population.

## 5. Conclusions

Our study revealed a prevalent occurrence of neophobia both among patients diagnosed with PKU and the control group. Among PKU patients, neophobia exhibited correlations with various factors, including the patient’s age, the time lapse between birth and disease diagnosis, and the educational attainment of the parents. These findings underscore the multifaceted nature of neophobia in individuals with PKU, shedding light on potential factors influencing its prevalence and manifestation. Also, by correlating the educational level of the parent or carer with neophobia incidence, we believe that offering clear and strong information to parents at the time of diagnosis and providing nutritional guidance can decrease the occurrence of this eating behavior disorder. The study also concluded that diagnosing as soon as feasible after birth and implementing a suitable diet are factors that are correlated with reducing the occurrence of neophobia. 

## Figures and Tables

**Table 1:** Statistical description of the lot and results analyses.

Parameters	PKUG (*n* = 34)	CG (*n* = 34)	*p*-Value
Sex % (*n*)			
Males	67.6 (23)	67.6 (23)	1
Females	32.4 (11)	32.4 (11)	
Age at diagnosis in days (median, IQR)	21(15, 41)		
Age in years (median, IQR)	7.05 (4.9, 15.2)	5.75 (3.8, 14.87)	0.615

Abbreviations: PKUG, phenylketonuria group; CG, control group; IQR, inter-quartile range.

**Table 2:** *PAH* gene mutations and phenotypic associations.

N0.	*PAH* Variant	Zygosity Status	ACMG [30] Classification	PKU Phenotype
001	NM_000277.3:c.1066-11G>A	Heterozygous	Pathogenic	Classic or moderate
001	NM_000277.3:c.1222C>T NP_000268.1:p.(Arg408Trp)	Heterozygous	Pathogenic	Classic
002	NM_000277.3:c.472C>T NP_000268.1:p.Arg158Trp	Heterozygous	Pathogenic	Classic
002	NM_000277.3:c.1222C>T NP_000268.1: p.Arg408Trp	Heterozygous	Pathogenic	Classic
003	NM_000277.3:c.1222C>T NP_000268.1: p.Arg408Trp	Homozygous	Pathogenic	Classic
004	NM_000277.3:c.1222C>T NP_000268.1:p.Arg408Trp	Homozygous	Pathogenic	Classic
005	NM_000277.3:c.754C>T NP_000268.1: p.Arg252Trp	Heterozygous	Pathogenic	Classic
005	NM_000277.3:c.782G>A NP_000268.1:p.Arg261 GLN	Heterozygous	Pathogenic	Classic or moderate
006	NM_000277.3:c.1222C>T NP_000268.1:p.Arg408Trp	Heterozygous	Pathogenic	Classic
006	NM_000277.3:c.782G>A NP_000268.1:p.Arg261Gln	Heterozygous	Pathogenic	Classic or moderate
007	NM_000277.3:c.1222C>T NP_000268.1:p.(Arg408Trp)	Homozygous	Pathogenic	Classic
008	NM_000277.3:c.1222C>T NP_000268.1:p.Arg408Trp	Homozygous	Pathogenic	Classic
009	NM_000277.3:c.1315+1G>A	Heterozygous	Pathogenic	Classic
009	NM_000277.3:c.533A>G NP_000268.1:p.Glu178Gly	Heterozygous	Pathogenic	Mild
010	NM_000277.3:c.1222C>T NP_000268.1:p.(Arg408Trp)	Homozygous	Pathogenic	Classic
011	NM_000277.3:c.1222C>T NP_000268.1:p.(Arg408Trp)	Homozygous	Pathogenic	Classic
012	NM_000277.3:c.1222C>T NP_000268.1:p.(Arg408Trp)	Homozygous	Pathogenic	Classic
013	NM_000277.3:c.1222C>T NP_000268.1:p.(Arg408Trp)	Homozygous	Pathogenic	Classic
014	NM_000277.3:c.1222C>T NP_000268.1:p.(Arg408Trp)	Homozygous	Pathogenic	Classic
015	NM_000277.3:c.1222C>T NP_000268.1:p.(Arg408Trp)	Homozygous	Pathogenic	Classic
016	NM_000277.3:c.1222C>T NP_000268.1:p.(Arg408Trp)	Homozygous	Pathogenic	Classic
017	NM_000277.3:c.1222C>T NP_000268.1:p.(Arg408Trp)	Homozygous	Pathogenic	Classic

Abbreviations: N0., patient ID; ACMG, American College of Medical Genetics and Genomics guidelines; PKU, phenylketonuria.

**Table 3:** Tertiles of FNS score results for both study groups.

Age Group (Years)	Group and Sex	FNS < 30 (%)	31–35 FNS (%)	>35 FNS (%)
Under 2	PKUG—male	33.33	33.33	33.33
	PKUG—female	0.00	0.00	0.00
	Total PKUG	33.33	33.33	33.33
	CG male	33.33	0.00	66.67
	CG female	0.00	0.00	0.00
	Total CG	33.33	0.00	66.67
2–7.9	PKUG—male	18.18	9.09	72.73
	PKUG—female	25.00	25.00	50.00
	Total PKUG	14.29	14.29	71.43
	CG male	0.00	8.33	91.67
	CG female	0.00	0.00	100.00
	Total CG	0.00	6.67	93.33
8–13.9	PKUG—male	0.00	0.00	100.00
	PKUG—female	0.00	50.00	50.00
	Total PKUG	0.00	16.67	83.33
	CG male	0.00	0.00	100.00
	CG female	0.00	0.00	100.00
	Total CG	0.00	0.00	100.00
14–17.9	PKUG—male	0.00	25.00	75.00
	PKUG—female	50.00	0.00	50.00
	Total PKUG	16.67	16.67	66.67
	CG male	25.00	0.00	75.00
	CG female	0.00	0.00	100.00
	Total CG	16.67	0.00	83.33
Over 18	PKUG—male	0.00	0.00	100.00
	PKUG -female	0.00	0.00	100.00
	Total PKUG	0.00	0.00	100.00
	CG male	0.00	0.00	100.00
	CG female	0.00	0.00	100.00
	Total CG	0.00	0.00	100.00

Abbreviations: PKUG, phenylketonuria group; CG, control group; FNS, Food Neophobia Scale.

**Table 4:** PKUG in comparison with CG statistical analyses.

Parameters	PKUG (*n* = 34)	CG (*n* = 34)	*p*-Value
Development median (IQR)	80 (43.7, 99.9)	75.5 (22.5, 97)	0.473
Primary caretaker % (*n*)			0.003
Mother	91.2 (31)	41.2 (14)	
Other	8.8 (3)	58.8 (20)	
Mother education % (*n*)			0.634
Primary school	23.5 (8)	23.5 (8)	
Vocational school	14.7 (5)	14.7 (5)	
High school	14.7 (5)	26.5 (9)	
University	47.1 (16)	35.2 (12)	
Father education % (*n*)			0.279
Primary school	17.6 (6)	20.6 (7)	
Vocational school	17.6 (6)	38.2 (13)	
High school	23.5 (8)	14.7 (5)	
University	41.2 (14)	26.5 (9)	
FNS median (IQR)	38.5 (30, 46)	45 (40, 49)	0.013
Level of Phe at birth median (IQR)	1089.6 (484.3, 1785.8)		
Level of Phe at present median (IQR)	260.3 (183.4, 381.3)		

Abbreviations: PKUG, phenylketonuria group; CG, control group; FNS, Food Neophobia Scale. Phe levels are measured in µmol/L. Statistically significant differences, with *p* < 0.05, are represented in bold.

**Table 5:** FNS score based on the environment of origin (rural/urban).

Independent Samples *t*-Test Rural/Urban	W	*p*-Value	Hodges–Lehmann Estimate	Rank-Biserial Correlation
FNS	141.000	0.931	−3.769 × 10^–5^	−0.021
Birth weight (g)	146.500	0.945	2.580 × 10^–5^	0.017
Birth height (cm)	157.500	0.651	1.000	0.094
Current age (months)	134.500	0.756	−4.331	−0.066
BMI	155.000	0.717	0.330	0.076
Time to diagnosis (years)	145.000	0.986	4.815 × 10^–6^	0.007
Age at diagnosis (months)	157.500	0.653	2.685	0.094
Level of Phe at birth (µmol/L)	107.500	0.313	−5.000	−0.210
Current level of Phe (µmol/L)	126.500	0.772	−0.500	−0.063
Current weight (kg)	152.000	0.796	1.577	0.056
Current height (cm)	154.000	0.743	2.451	0.069

Abbreviations: FNS, Food Neophobia Scale; BMI, body mass index; Phe, phenylalanine.

**Table 6:** The results of tested hypotheses.

No	Hypothesis	Result
H1	There is a variation of distribution for FN score among PKU group and control group.	Rejected
H2	There is a relationship between the patients’ current BMI and the development of neophobia.	Rejected
H3	There is a relationship between diet compliance and neophobia.	Rejected
H4	There is a relationship between a patient’s Phe level at the diagnostic moment and the development of neophobia.	Rejected
H5	There is a correlation between parental educational level and the patient’s development of neophobia.	Accepted
H6	There is a correlation between the patient’s age and the onset of neophobia.	Accepted
H7	There is a relationship between length of period from birth to diagnostic and neophobia.	Accepted
H8	There is a variation of distribution for FN score among urban and rural patients.	Rejected

## Data Availability

The data presented in this study are available on request from the corresponding author. The data are not publicly available due to ethical restrictions.

## Supplementary Materials

The following supporting information can be downloaded at https://www.mdpi.com/article/10.3390/nu16060768/s1: Table S1: Adapted version of the Food Neophobia Scale [7].
