# Supplementary material for: Factors Impacting the Reduction in Neophobia Prevalence in Phenylketonuria Patients
Source: Nutrients. 2024 Mar 7;16(6):768. doi: 10.3390/nu16060768 (PMC10975818; doi:10.3390/nu16060768)
Supplement: Supplementary file 1 [file nutrients-16-00768-s001.zip › nutrients-2888355-supplementary.pdf]

Q1	The child has the curiosity to try new foods
Q2	The child does not trust newly introduced foods
Q3	If he doesn't know the ingredients that are in the food, the child doesn't try it
Q4	The child likes to try food specific to other countries
Q5	Foods with unknown specific look weird to be eaten
Q6	The child tries new foods when at parties
Q7	The child is afraid to try new foods, unconsumed before
Q8	The child is very clear when it comes to the foods he wants to eat
Q9	The child eats anything
Q10	The child would like to try new dishes from restaurants with different specialties
